# Long-Gauge Fiber Optic Sensors: Strain Measurement Comparison for Reinforced Concrete Columns

**DOI:** 10.3390/s25010220

**Published:** 2025-01-02

**Authors:** Haoran Lin, Zhaowen Xu, Wan Hong, Zhihong Yang, Yixin Wang, Bing Li

**Affiliations:** 1 School of Civil and Environmental Engineering, Nanyang Technological University, Singapore 639798, Singapore; haoran003@e.ntu.edu.sg (H.L.); cbli@ntu.edu.sg (B.L.); 2 Institute for Infocomm Research, Agency for Science, Technology and Research (A*STAR), 1 Fusionopolis Way, Singapore 138632, Singapore; zxu@i2r.a-star.edu.sg (Z.X.); wangyx@i2r.a-star.edu.sg (Y.W.); 3 College of Civil Engineering, Nanjing Tech University, Nanjing 211816, China; w.hong@njtech.edu.cn

**Keywords:** structural health monitoring, long-gauge fiber optic sensors, LVDT, point-sensor, eccentricity, RC column

## Abstract

Long-gauge fiber optic sensors have proven to be valuable tools for structural health monitoring, especially in reinforced concrete (RC) beam structures. While their application in this area has been well-documented, their use in RC columns remains relatively unexplored. This suggests a promising avenue for further research and development. This paper presents a thorough comparison of long-gauge fiber optic sensors and traditional measurement tools when used to monitor RC columns under small eccentric compressive loading. The evaluation focuses on the stability and precision of each sensor type. A monitoring system was developed for laboratory testing to assess the performance of various sensor types under specific conditions. The system incorporated four measurement schemes, utilizing a combination of embedded and surface-mounted long-gauge fiber optic sensors, linear variable differential transformers (LVDTs), and point sensors (strain gauges). Long-gauge fiber optic sensors, securely mounted on the concrete surface near the tensile side, were found to accurately measure both large and small deformations, outperforming LVDTs. Compared to strain gauges and embedded optic sensors, the long-gauge fiber optic sensors demonstrated superior average strain measurement and minimal interference from protective covers.

## 1. Introduction

Long-gauge fiber optic sensors are an advanced technology for detecting damage within a specific range. They provide significant benefits in challenging environments characterized by high conductivity and electromagnetic interference (EMI). These sensors offer exceptional performance and reliability in conditions that would be detrimental to traditional sensing methods [1]. Due to their ability to provide accurate and reliable measurements under diverse loading conditions, long-gauge fiber optic sensors have emerged as a promising technology for structural health monitoring (SHM). These sensors have gained significant attention in recent years for monitoring and assessing damage in various civil infrastructures, including large-scale structures like long-span bridges [2,3] and high-rise buildings [4]. They offer the potential to provide valuable insights into the structural health and behavior of both large- and small-scale structures [5]. For instance, Cheng et al. [6] evaluated the residual strength of corroded reinforced concrete (RC) beams using long-gauge length sensors and point-sensors. Xu et al. [7] developed a damage detection algorithm for RC beams. Long-gauge fiber optic sensors can indirectly predict deflection distribution in RC structures. Shen [8] and Li [9] applied and refined the conjugated beam method (CBM) for predicting deflection in elastic-stage RC beams. Hong [10] and Lin [11] extended CBM to account for nonlinearity and shear deformation.

A review of previous studies on small-scale RC structures reveals a predominance of research focusing on beam structures, with limited investigations into the performance of long-gauge fiber optic sensors on RC columns. This discrepancy may be attributed to the shorter height of columns compared to beam spans, suggesting that traditional point-sensors might suffice. Furthermore, the compressive strains on RC columns subjected to small eccentric loading often remain ambiguous throughout the loading process, hindering the reliable measurement of strain data using long-gauge fiber optic sensors. While long-gauge fiber optic sensors have been successfully applied to columns under seismic conditions [12] or impact loads [13], where strains are relatively large, their use in RC columns under minor strain conditions remains underexplored. Notably, studies by Glisic and Inaudi [14] have demonstrated the capability of long-gauge fiber optic sensors to accurately capture minor strain changes caused by shrinkage, creep, and temperature variations. However, these studies were constrained by limited sample sizes and budget restrictions, preventing a comprehensive evaluation of their performance in RC columns. To establish their reliability in this context, further experimental investigations with robust datasets are essential.

This study evaluated the effectiveness of long-gauge fiber optic sensors for short-term structural health monitoring of reinforced concrete columns under eccentric loading. To assess their accuracy, four identification methods were compared:

**Method 1** Surface-mounted Optic Sensors: Long-gauge fiber optic sensors were attached directly to the concrete surface on both the compressive and tensile sides.
**Method 2** Embedded Optic Sensors: Long-gauge fiber optic sensors were embedded within the concrete and attached to reinforcing bars, protected by a cover.
**Method 3** LVDTs: Linear variable differential transformers were placed on the same concrete surface as the surface-mounted optic sensors in Method 1.
**Method 4** Point-sensors: Strain gauges were attached to embedded reinforcing bars, in alignment with the embedded fiber optic sensors described in Method 2.

To determine the optimal sensor length for long-gauge fiber optic sensors, this study also compared measurement results from sensors of 30 mm, 60 mm, and 120 mm. Additionally, the reliability of these methods was assessed by comparing predicted bending moments and eccentricities to measured values.

## 2. Theoretical Background

### 2.1. Principle of Fiber Optic Sensor

**Figure 1:**
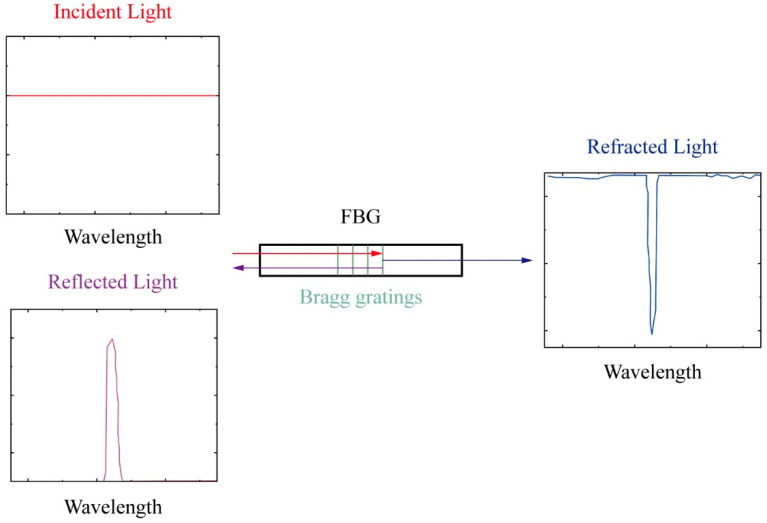
Schematic diagram of fiber Bragg grating [18].

The optic measurement system for structural health monitoring consists of a stable optical source, a measurement unit, a data acquisition system, and the necessary connections [15]. The optical source and data acquisition are integrated within the interrogator, while the fiber optic sensor functions as the measurement unit [16]. When the interrogator emits light at a specific wavelength, a portion of the light is reflected upon reaching the fiber gratings, while the remaining light passes through the fiber. As shown in Figure 1, the working principle of optic fiber sensors can be formulated as follows [17]:

(1) $$\lambda =2n_{eff}\Lambda$$

where λ is the Bragg wavelength and $n_{eff}$ and Λ are the refractive index and the intervals between gratings, respectively.

As discussed in Equation (1), both the variations in the refractive index and the microstructures of the fiber Bragg grating (FBG) can affect the output wavelength. Therefore, fiber optic sensors are highly sensitive to changes in strain (Δ*ε*) and temperature (Δ*T*), which can lead to the elongation or shortening of the FBG intervals [19] and changes in optical properties [20,21], respectively. Their influences can be expressed by the following equations:

(2) $$\;\frac{\Delta \lambda }{\lambda }=k\cdot \Delta \varepsilon$$

(3) $$\frac{\Delta \lambda }{\lambda }=\left(\alpha +\xi \right)\cdot \Delta T$$

where k is the Bragg grating factor accounting for the dependence of the refractive index on axial tension/compression loading condition and α and ξ are the expansion and thermos-optic coefficients, respectively.

Fiber optic sensors offer several advantages over traditional sensors, including their ability to be influenced solely by temperature and strain, as well as their multiplexing capability. Multiplexing enables multiple sensors to operate on a single optical fiber, simplifying the deployment of long-gauge fiber optic sensors in RC structures. Furthermore, FBG sensors exhibit minimal light intensity loss during signal transmission [22], which ensures the foundation for accurate measurements by distributed long-gauge fiber optic sensors. Despite the drawbacks of high cost and susceptibility to fracture, these sensors remain suitable for measuring compressive strain on RC columns under quasi-static loading conditions.

### 2.2. Algorithm for Determining the Bragg Wavelength

**Figure 2:**
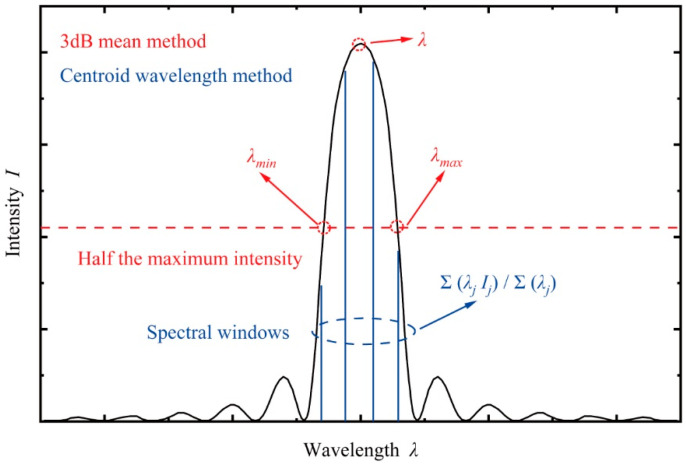
3 dB mean method and centroid wavelength method for identifying the peak Bragg wavelength.

To identify the peak Bragg wavelength reflected by an FBG, the spectrum must be processed using appropriate algorithms. The most widely used methods in interrogators with scanning sources are the 3 dB mean method and the centroid wavelength method [23], as illustrated in Figure 2. The 3 dB mean method calculates the mean of the wavelengths corresponding to the two data points whose intensities are closest to half the maximum intensity. The calculation is expressed as:

(4) $$\lambda =\frac{\lambda_{min}+\lambda_{max}}{2}$$

where $\lambda_{min}$ and $\lambda_{max}$ denote the minimum and maximum wavelength at half the maximum intensity, respectively. This method is computationally efficient due to its simplicity, making it suitable for fast processing. However, it is less effective for spectra with asymmetric shapes, where inaccuracies may occur.

The centroid wavelength method is based on a mathematical definition, which calculates the weighted average of wavelengths using their corresponding intensities. The formula is given as:

(5) $$\lambda =\frac{\sum_{j=1}^{n}\;\lambda_{j}I_{j}}{\sum_{j=1}^{n}I_{j}}$$

where $\lambda_{j}$ is the measured wavelength and $I_{j}$ is the corresponding reflected intensity at the *j*-th spectral window. This method is well-suited for applications requiring coarse resolution measurements but necessitates multiple spectral windows for accurate results.

### 2.3. Predictions of Bending Moment and Eccentricity

**Figure 3:**
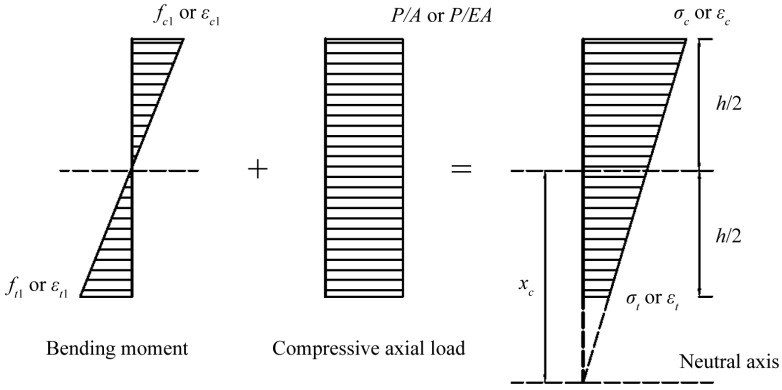
Stress (strain) diagram: superposition of stresses (strains) from axial load and bending moment.

The stresses acting on the cross-section of the RC column result from the superposition of axial load and bending moment, as illustrated in Figure 3. Due to the small eccentricity, the entire cross-section experiences compressive stress, causing the neutral axis to lie outside the cross-section. Based on the strain readings from sensors positioned on the “compressive” and “tensile” sides of the concrete surface, the neutral axis position, $x_{c}$, is calculated using the relationship in stress (strain) diagram:

(6) $$x_{c}=\frac{\left(\bar{\sigma_{c}}+\bar{\sigma_{t}}\right)}{2(\bar{\sigma_{c}}-\bar{\sigma_{t}})}h=\frac{\left(\bar{\varepsilon_{c}}+\bar{\varepsilon_{t}}\right)}{2(\bar{\varepsilon_{c}}-\bar{\varepsilon_{t}})}h$$

where $\bar{\sigma_{c}}$($\bar{\varepsilon_{c}}$) and $\bar{\sigma_{t}}$($\bar{\varepsilon_{t}}$) are the average “compressive” and “tensile” stress (strain), respectively, and h denotes the height of the cross-section.

The bending moment, *M*, and eccentricity, e, can be derived using the following expression [24]:

(7) $$M=Pe=\frac{E_{c}\cdot \bar{\varepsilon_{c}}\cdot I}{\frac{h}{2}+x_{c}}$$

where P is the applied compressive axial load, $E_{c}$ is the elastic modulus of concrete (estimated as 5000$\sqrt{f_{c}^{\prime }}$), and I is the moment of inertia of the cross-section. For simplicity, only the moment of inertia of the concrete is considered (calculated as $I=\frac{1}{12}bh^{3}$), where b is the width of the cross-section.

By substituting Equation (6) into Equation (7), the bending moment and eccentricity can be expressed in terms of the differences between the average “compressive” and “tensile” strains, as follows:

(8) $$M=Pe=\frac{bh^{2}}{12}E_{c}\left(\bar{\varepsilon_{c}}-\bar{\varepsilon_{t}}\right)$$

It is important to note that, in this equation, all values in the compressive direction should be taken as negative.

## 3. Experimental Investigation

### 3.1. Experimental Setup

**Figure 4:**
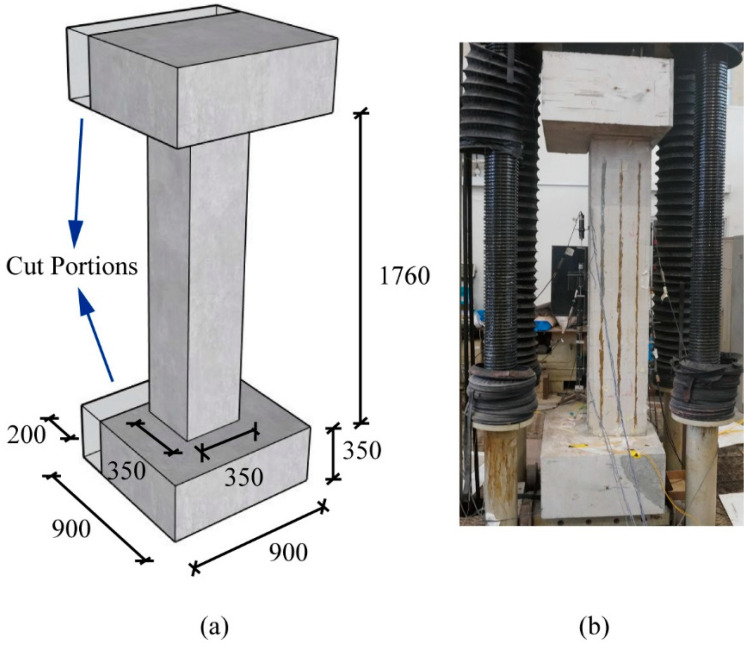
Detailed dimensions of the column structure C1: (**a**) in the diagram and (**b**) in the test.

To assess the accuracy of long-gauge fiber optic sensors, a reinforced concrete (RC) column structure, C1, was constructed. The column had a square cross-section of 350 mm × 350 mm and a height of 1760 mm. To facilitate the use of the IMU 5000 loading device, two concrete blocks measuring 200 mm × 900 mm × 350 mm were removed from both the top and bottom studs, as shown in Figure 4a. Consequently, the column in the test is subjected to loading with a small eccentricity, as shown in Figure 4b. 

**Figure 5:**
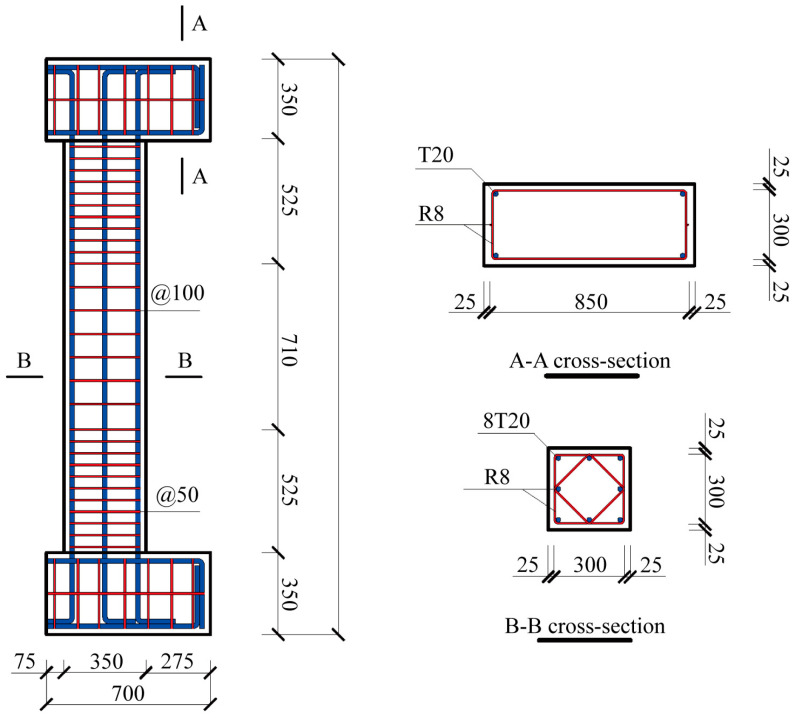
Reinforcement details for column structure C1.

Both the column and studs were fully reinforced with T20 longitudinal bars and R8 transverse bars. Figure 5 details the reinforcement of the column, with T20 longitudinal bars distributed along the edge of the cross-section and R8 transverse bars placed vertically and obliquely to ensure shear capacity by forming four stirrups in each cross-section. Tensile tests revealed that the yield strengths of the T20 and R8 bars were 580 MPa and 286 MPa, respectively, with a 20% hardening. Similarly, the average compressive strength of the concrete was measured at 40 MPa.

**Figure 6:**
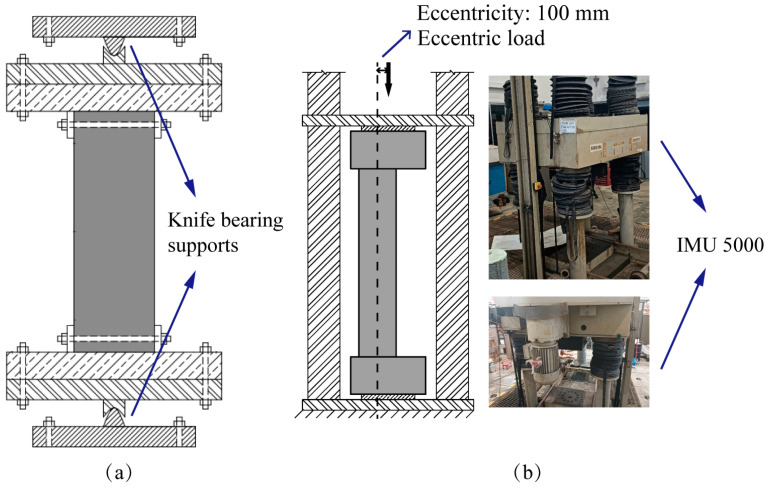
Loading schemes for columns under eccentric loading: (**a**) with knife bearing supports [25] and (**b**) without knife bearing supports.

When applying eccentric loading, gaps between the cross-sectional plane and the loading plate are inevitable due to the influence of bending moments. These gaps can cause the applied loads to deviate from being perpendicular to the cross-sectional plane, resulting in uneven load distribution from the loading plates and introducing random eccentricity. To address this issue, it is essential to use knife-edge bearing supports (Figure 6a [25]), which ensure that the applied load remains perpendicular to the cross-sectional plane, thereby allowing precise control of eccentricity. However, due to constraints imposed by the available instrumentation during the laboratory test, the loading scheme depicted in Figure 6b was implemented. Consequently, the column structure C1 was subjected to random eccentricity loads. To accurately determine the actual eccentricity, Equation (8) in Section 2.3 was utilized.

### 3.2. Monitor System

**Figure 7:**
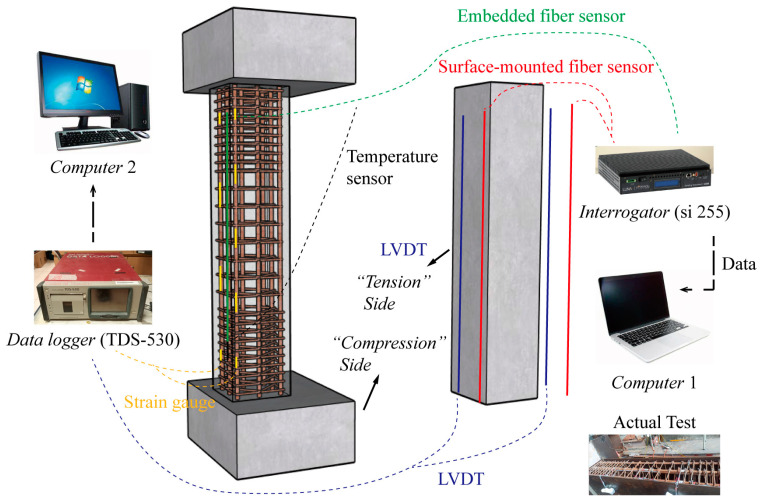
Proposed monitoring system for RC column.

The compressive behavior of the RC column was investigated using four distinct sensor types: bare fiber optic sensors (with and without packaging), Linear Variable Differential Transformers (LVDTs), and strain gauges. A monitoring system was developed as shown in Figure 7, where embedded long-gauge fiber optic sensors and strain gauges were embedded within the column, while surface-mounted fiber optic sensors and LVDTs were distributed along the concrete surface. A data logger (TDS-530) was used to collect results from the LVDTs and strain gauges, offering a measurement range of 40,000 με and a maximum measurement accuracy of 1 με. An interrogator (si255) with a 1 pm wavelength resolution and a 10 Hz data rate was used to precisely record the output from the fiber optic sensors. The actual test setup is also provided in Figure 7.

### 3.3. Layout of Sensors

**Figure 8:**
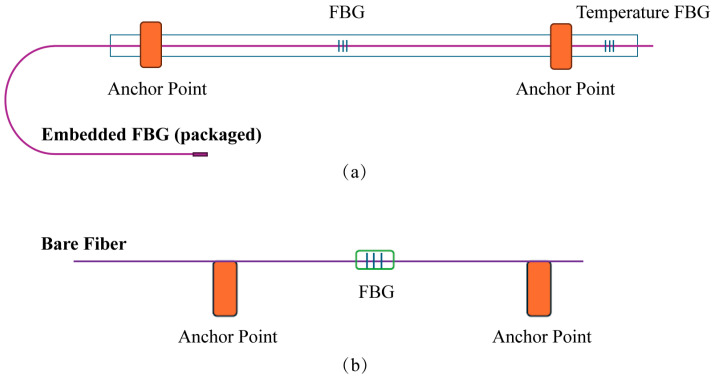
Configurations of (**a**) embedded FBG sensor (packaged) and (**b**) bare FBG sensor.

**Figure 9:**
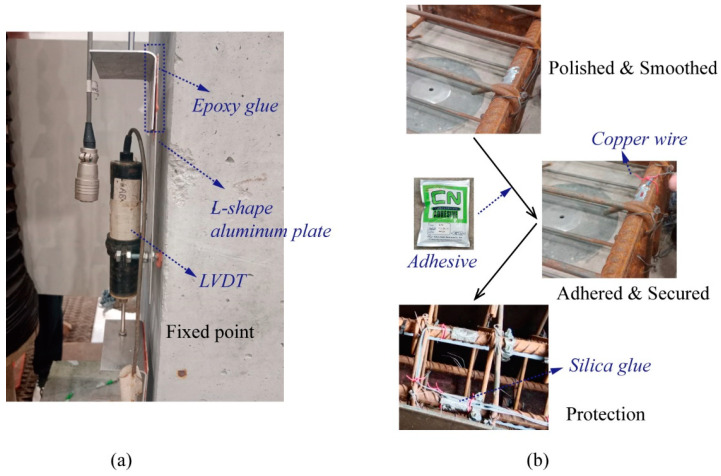
Installations of (**a**) LVDTs and (**b**) strain gauges.

To protect the delicate sensors and prevent unexpected failures during the structural monitoring of the RC column, those following approaches were used:

(1) Bare optical fiber is inherently fragile and requires adequate protection. To achieve this, the FBG sensor was enclosed within a protective PA12 tube, with the fiber securely anchored at two points using epoxy. As shown in Figure 8a,b for embedded and surface-mounted sensors, respectively, each anchoring point comprised a steel structure attached to the PA12 tube, featuring a small hole through which the fiber passed. After the epoxy cured, the sensor was firmly fixed in position. The gauge length between the two anchoring points was carefully designed to match the dimensions of the test structure. The strain FBG sensor was centrally located between the two anchoring points, while the temperature FBG sensor was positioned at the end of the long-gauge assembly. This arrangement ensured both precise measurements and adequate protection for the optical fibers throughout the testing process.
(2) Figure 9 illustrates the installation of LVDTs and strain gauges. Target points are required for non-contact LVDTs to measure deformation at specific distances. L-shaped aluminum plates, as shown in Figure 9a, are used to provide these target points. Both the LVDTs and L-shaped aluminum plates are secured to the column surface with bolts. Strain gauges must be firmly attached to the rebar to accurately detect deformation. Figure 9b depicts the installation process for strain gauges: First, the rebar surface at designated positions is polished and smoothed. Then, the strain gauges are adhered and secured to the rebar surface using adhesives and copper wire. Finally, epoxy glue is applied to complete the installation.

**Figure 10:**
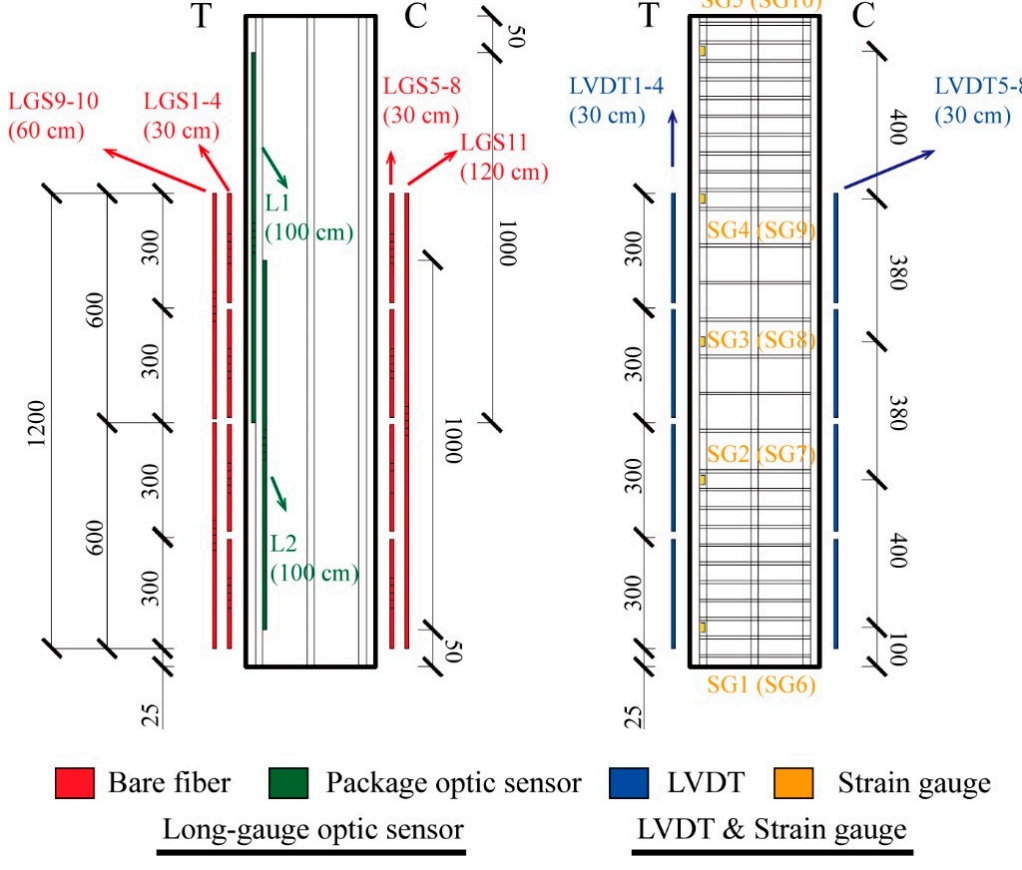
Arrangement of long-gauge sensors, strain gauges, and LVDTs along the reinforcement bars or concrete surface in the RC column structure C1.

The layouts of long-gauge fiber optic sensors and other sensors (strain gauges and LVDTs) in the RC column C1 are illustrated in Figure 10. Two embedded long-gauge fiber optic sensors (L1 and L2), each with a length of 100 cm, were embedded on the tensile sides of the column. Additionally, fiber optic sensors with lengths of 30 cm (LGS1–8), 60 cm (LGS9–10), and 120 cm (LGS11) were mounted on the concrete surface to investigate the optimal length for long-gauge fiber optic sensors. Theoretically, the stress on the eccentric-loaded column is independent of its height. For ease of installation, these optical sensors were mounted starting from the bottom and extending up to 100 cm. Calibration of the optical sensors to ensure a linear relationship between strain and wavelength was performed using a motion controller (ESP 300), as detailed in Table 1. Consequently, the corresponding strain values can be determined based on the changes in measured wavelengths. LVDTs (LVDT1–8) were also installed on the concrete surface in alignment with the 30 cm surface-mounted long-gauge optical sensors, while strain gauges (SG1–10) were evenly distributed on the steel bars on the “tensile” sides, corresponding to the embedded optical sensors.

## 4. Results and Discussion

### 4.1. Experimental Results

Before conducting the test, it is essential to pre-tension the bare fiber optic sensors, since the column primarily experiences compressive stress during the experiment. The process involves securing one anchor point and carefully stretching the other. During this stretching, the wavelength of the strain sensor is continuously monitored using the FBG interrogator. When the wavelength reaches the desired value, indicating the required level of pre-tension, the second anchor point is fixed to lock the sensor in place. Numerical simulations through finite element analysis software have indicated that an optimal pre-strain of 5000 micro-strains should be applied to all fiber optic sensors.

Column specimen C1 underwent a quasi-static loading scheme. As depicted in Figure 6, a geometric eccentricity of 100 mm was introduced, though the actual eccentricity during the test remains uncertain. Given the high axial stiffness of the RC column, the loading protocol began with force-controlled increments of 100 kN. Once a substantial reduction in the column’s stiffness was detected, the loading mode transitioned to displacement-controlled, continuing until the end of the test.

**Figure 11:**
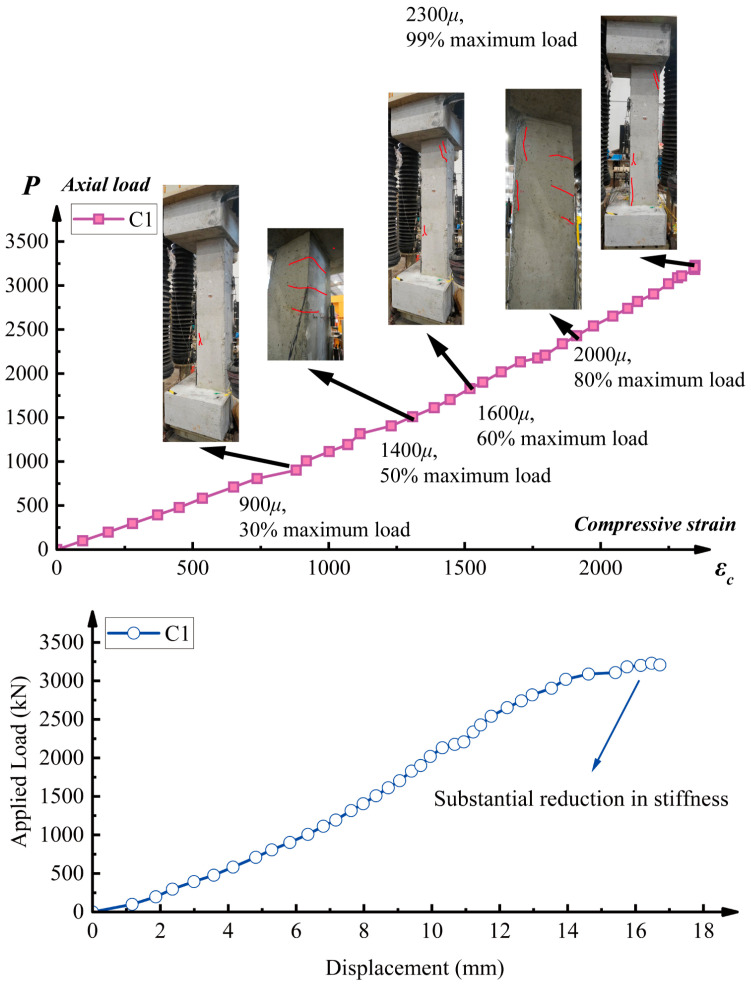
Axial load/compressive strain curve (average compressive strain from LGS5 to LGS8), load-displacement curve, and crack pattern for column specimen C1.

As depicted in Figure 11, the load versus axial strain curve and crack pattern for column specimen C1 reveal characteristic behavior. Due to the limited development of the tensile zone, the specimen exhibited a relatively low density of surface cracks. The initial crack appeared at a load of 1600 kN. As the applied load increased, existing cracks extended, but their width remained relatively constant. A significant reduction in column stiffness was observed when the load approached 3200 kN, coinciding with the formation of a primary vertical crack on the “tensile” side. To mitigate the risk of sudden failure, the test was terminated after implementing several displacement-controlled steps.

### 4.2. Comparisons of Sensors and Measurement Schemes

**Figure 12:**
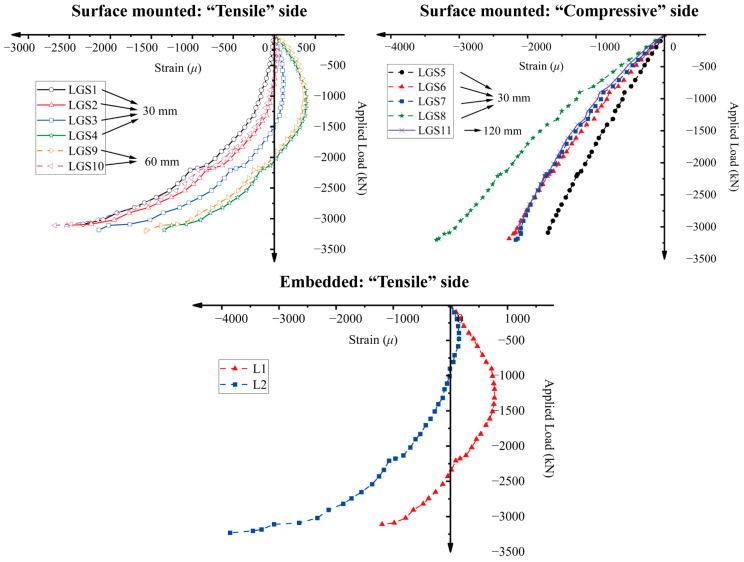
Load–strain curves for surface-mounted “tensile” side, surface-mounted “compressive” side, and embedded “tensile” side long-gauge fiber optic sensors.

**Figure 13:**
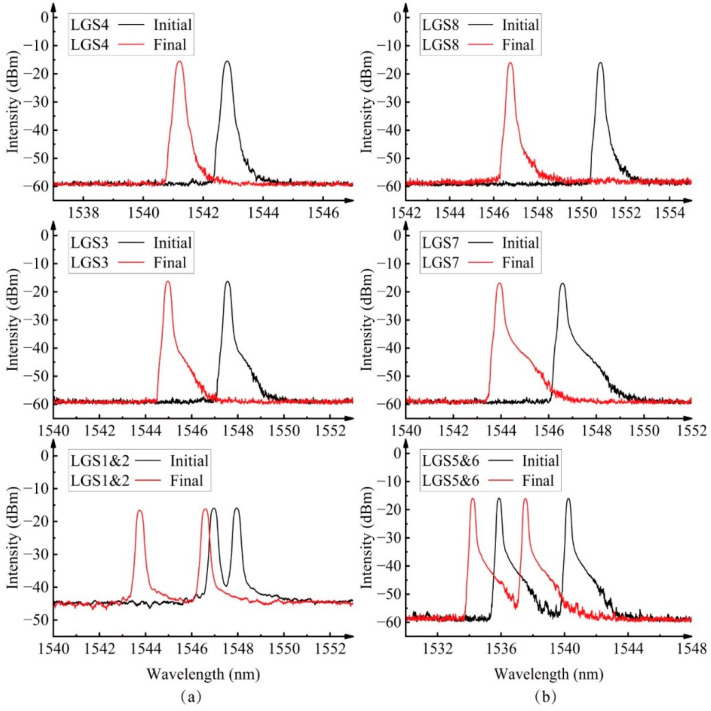
Initial and final spectra for fiber optic sensors on the (**a**) “tensile” side and (**b**) “compressive” side.

Real-time monitoring of the RC column was conducted using long-gauge sensors, with the load–strain curves for all optical sensors displayed in Figure 12. Sensors LGS1–4, LGS9–10, and L1–2 were installed on the “tensile” side. Due to the tensile strain induced by bending being counteracted by the compressive strain from axial loading, the increase in compressive strain on this side was relatively slow. Sensors LGS3, LGS4, and LGS6, located in the middle section of the column, initially experienced tensile strain due to the formation of micro-cracks. These cracks compressed the concrete at the column’s end, preventing any tensile strain from being recorded by sensors LGS1, LGS2, and LGS5, which were installed near the column’s end. The load–strain curves for embedded long-gauge sensors L1 and L2 exhibited a similar trend. The compressive strains of LGS5–8 showed a rapid increase due to the combined effects of bending and axial loading. Figure 13 compares the initial and final spectra for both tensile and compressive side sensors with a 30 cm gauge length. All sensors exhibited a decrease in wavelengths after the test, indicating a final state of compression. While the formation of cracks initially introduced tensile strains in certain areas of the small eccentric axial compression column, the inability of these cracks to propagate in the highly compressed environment prevented the development of sustained tensile strains within the column at its maximum load-bearing capacity.

**Figure 14:**
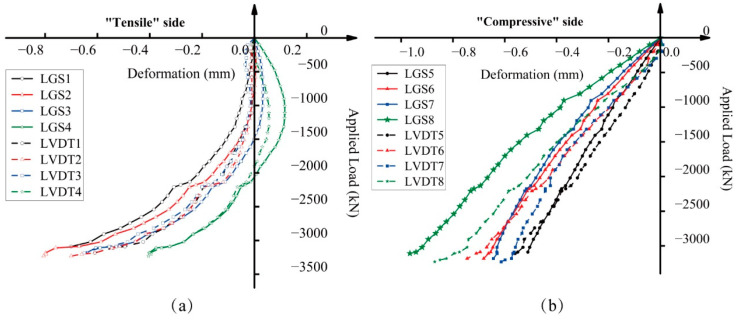
Comparisons of strain measurements from long-gauge fiber optic sensors and LVDTs: (**a**) “tensile” side and (**b**) “compressive” side.

As illustrated in Figure 10, LVDT1–8 were positioned on both the “tensile” and “compressive” sides, corresponding to LGS1–8. To verify the accuracy of the measurements from long-gauge fiber optic sensors, Figure 14 presents a comparison between these two types of measurement tools. The curves from the long-gauge fiber optic sensors demonstrated excellent agreement with those from the LVDTs, with errors generally within 15%. This validates the measurement accuracy of long-gauge sensors in small eccentric loaded columns. Moreover, the analysis of the results reveals that long-gauge fiber optic sensors (LGSs) exhibit greater stability in detecting large strains and higher precision in detecting small strains compared to traditional LVDTs. For example, in Figure 14b there is a significant discrepancy between LGS8 and LVDT8. This instability in LVDT measurements could be attributed to the complex construction of the fixed point, as illustrated in Figure 9a. Errors in setup or vibrations from external environments can lead to inaccuracies in LVDT measurements. A similar significant error can be observed between LGS2 and LVDT2 in Figure 14a. However, this error is unavoidable since LVDTs can only detect deformations of 0.005 mm or larger. As a result, the readings from LVDT2 were approximately zero during the initial steps.

Furthermore, in practical engineering applications, identifying a fixed point for displacement transducers that remains free from external interference is a significant challenge, especially for long-term monitoring. While LVDTs with smaller measurement ranges (e.g., 3 mm) may offer better accuracy in measuring concrete strain and are generally cost-effective in most environments, their practical application in real-world reinforced concrete (RC) structures is often limited. Considering these factors, long-gauge fiber optic sensors are considered superior to traditional LVDTs due to their enhanced stability and higher precision.

**Figure 15:**
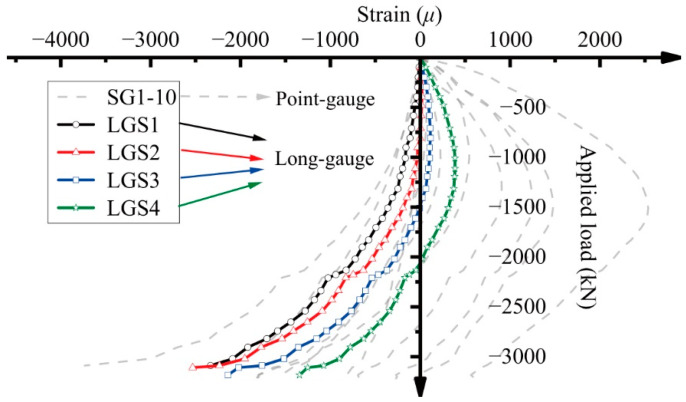
A comparison of strain measurements from long-gauge sensors on the “tensile” side and point-sensors (strain gauges).

The strain gauges (SG1–10) were uniformly distributed along the reinforced bars on the “tensile” side. Figure 15 compares the strains at specific locations recorded by the strain gauges with the average strains measured by the long-gauge fiber optic sensors on the “tensile” side. It is clear that the curves from the long-gauge fiber optic sensors are enveloped by those from the strain gauges. This is because strain gauges (point-sensors) can detect extreme strain values, whereas long-gauge fiber optic sensors only capture the average strain over a certain distance. Thus, if the most likely location of damage can be identified in advance, point-sensors are the preferred choice. Otherwise, long-gauge sensors (LGSs) not only reduce the number of sensors required for structural health monitoring but also help determine the approximate location of damage based on changes in average strain.

**Figure 16:**
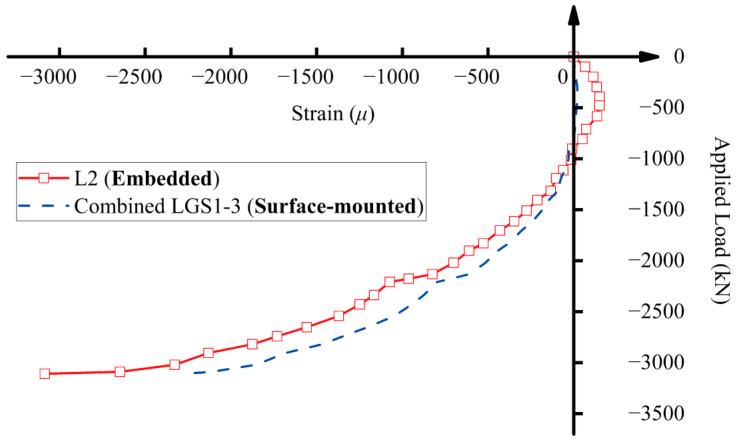
A comparison of average strains from embedded long-gauge fiber optic sensor L2 and combined bare optic fibers LGS1, LGS2, and LGS3.

As shown in Figure 10, L1 and L2 were embedded long-gauge fiber optic sensors that were securely attached to the surface of the rebar. The location of L2 generally corresponded to the positions of LGS1, LGS2, and LGS3. A comparison of the average strains between L2 and the combined measurements of LGS1, LGS2, and LGS3 is presented in Figure 16. The curves from these two setups indicate that, with proper installation, the protective cover has minimal impact on the measurement results of long-gauge fiber optic sensors. Furthermore, the protective cover effectively safeguards the sensors from potential damage caused by external factors outside the experiment. Therefore, in practical applications, it is essential to package the optic sensors to ensure their durability and reliability.

**Figure 17:**
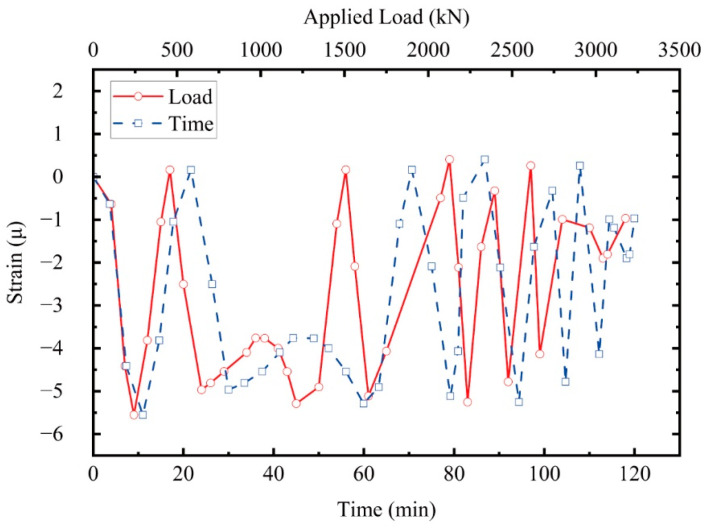
Temperature compensation (strain versus load and time) during the test.

It should be noted that the above conclusions are based on temperature compensation, despite the fact that temperature changes during the short-term tests were not significant, as shown in Figure 17. Over the two-hour experiment, the variation in temperature strain ranged from −6 μ to 1 μ.

### 4.3. Study of the Optimized Gauge Length for Optic Sensors

**Figure 18:**
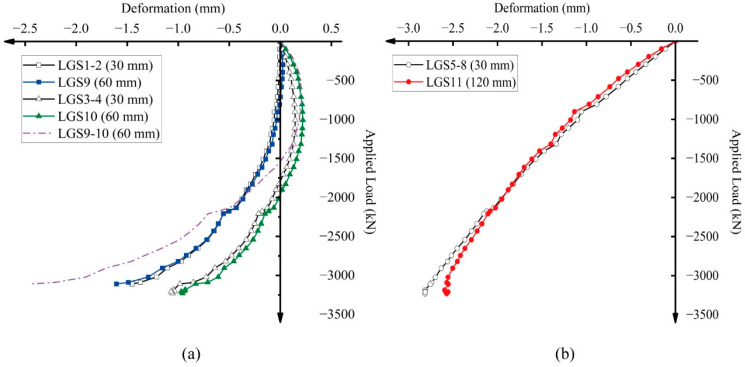
Comparisons of different lengths of long-gauge fiber optic sensors on the (**a**) “tensile” side and (**b**) “compressive” side.

To examine the influence of gauge length on the measured average strain, three types of long-gauge fiber optic sensors with lengths of 30 cm, 60 cm, and 120 cm [26] were installed on the “tensile” and “compressive” concrete surfaces, respectively. These sensors were positioned in close proximity to each other to minimize spatial variability. Due to budget constraints in instrumentation, the 60 cm optic sensors were only placed on the “tensile” side, while the 120 cm sensors were placed on the “compressive” side. As the measurement accuracy of the 30 cm optic sensors was validated through comparisons with the corresponding LVDTs, the optic sensors of other lengths can be directly compared with the measurement results of the 30 cm sensors, as shown in Figure 18.

The curves in Figure 18a for the 60 cm sensors and Figure 18b for the 120 cm sensors show strong agreement with those of the 30 cm optic sensors, suggesting that gauge length has a minimal impact on average strain measurement. However, as discussed in Section 4.2, the force mechanism on the “tensile” side is considerably more complex than on the “compressive” side. The stress state on the tensile side of the column transitions from compression to tension (C–T) due to the formation of micro-cracks. This transition occurs from the column end to the middle section. In such cases, optic sensors with an excessively long gauge length may not accurately capture the actual strain distribution along the column’s height. For example, the purple curve (LGS9–10) in Figure 18a is more influenced by the strain in the middle section of the column and fails to reflect the compression at the column end. On the compressive side, where the force mechanism is less complex, 120 cm optic sensors can be effectively used.

The optimal gauge length for optic sensors in RC structures is influenced by the loading conditions. For the small eccentrically loaded column (1780 mm) in this study, both 30 cm and 60 cm sensors were found to be suitable. However, future research may explore the potential impact of gauge length on measurement accuracy under different loading scenarios. These conclusions align with the findings of Glisic et al. [26].

### 4.4. Discussion of Predicted Bending Moment and Eccentricity

**Figure 19:**
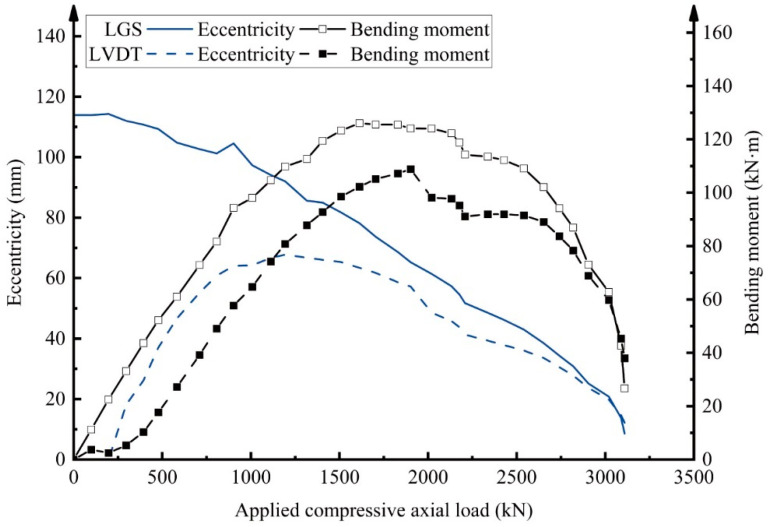
Estimation of bending moment and eccentricity via measurements from long-gauge fiber optic sensors (LGS1–4 and LGS5–8) and LVDTs (LVDT1–4 and LVDT5–8).

The shifts in bending moment and eccentricity of the RC column under small eccentric loading were predicted using Equation (8). The average “compressive” and “tensile” strains used in this equation were obtained from long-gauge fiber optic sensors or LVDTs installed on the respective sides of the column. As shown in Figure 19, the prediction results align extremely well with the experimental observations.

During eccentric loading, the column’s top experienced bending, causing it to rotate. Without the support of roller bearings, the loading plate remained stationary, leading to an uneven distribution of the applied load. This uneven distribution gradually shifted the load center from the “compressive” side to the “tensile” side, resulting in a decrease in eccentricity. Long-gauge fiber optic sensors confirmed this shift, measuring a reduction in eccentricity from approximately 110 mm to 10 mm. The trends observed in Figure 14 support this analysis, showing that, towards the end of the experiment, compressive strains on both the “compressive” and “tensile” sides were nearly equal. Given the increasing applied axial load and the significant reduction in eccentricity, the bending moment initially rose but subsequently decreased. This trend is likely due to the decreasing eccentricity and the increasing axial load, which together influenced the bending moment behavior. In comparing the estimations of eccentricity and bending moment, the superior performance of long-gauge fiber optic sensors over LVDTs in monitoring RC columns under small eccentric loading is further validated.

## 5. Conclusions

This paper presents a short-term structural health monitoring study on a reinforced concrete (RC) column subjected to eccentric loading using long-gauge optical sensors. A monitoring system was designed and implemented for laboratory testing. The system comprised embedded optical sensors, bare optical fibers, linear variable differential transformers (LVDTs), and strain gauges. Long-gauge fiber optic sensors were rigorously tested in a compressive environment, and their performance was compared to traditional measurement tools like LVDTs and strain gauges. The key findings are summarized as follows:

- Long-gauge fiber optic sensors offer several advantages over LVDTs for measuring both large and small deformations. Their distributed measurement approach eliminates the need for a fixed reference point, reducing the risk of measurement errors due to structural instability. Additionally, long-gauge fiber optic sensors can detect smaller strain changes with higher precision, making them ideal for applications requiring sensitive and reliable deformation monitoring.
- Long-gauge fiber optic sensors excel in measuring average strain across a specified distance, while strain gauges are more adept at capturing localized, extreme values. For long-span structures or when the exact critical point is uncertain, long-gauge sensors provide a distinct advantage by offering a comprehensive overview of strain distribution.
- With proper installation, protective covers have a minimal effect on the measurement accuracy of long-gauge fiber optic sensors. Additionally, they act as a safeguard, shielding the sensors from accidental damage during construction or operation.

The article also discusses the optimal gauge length for long-gauge fiber optic sensors and further validates their superior performance. Due to the complexity of the force mechanism on the “tensile” side, optic sensors with excessively long gauge lengths are shown to be inadequate for accurately capturing the strain distribution. Furthermore, long-gauge fiber optic sensors can accurately distinguish between the average “compressive” and “tensile” strains at the initial stages of loading, enabling correct predictions of the eccentricity and bending moment acting on the cross-section.

## Figures and Tables

**Table 1:** Calibration for long-gauge fiber optic sensor L1: $R^{2}$ = 0.99.

Deformation (μm)	Strain (με)	Measured Wavelength (nm)	Wavelength Shift (pm)
0	0	1542.15	0
600	583	1542.73	585
1100	1068	1543.31	1163
1600	1553	1543.89	1744
2100	2039	1544.47	2324
2600	2524	1545.05	2905
3100	3010	1545.64	3492
3600	3495	1546.23	4084
4100	3980	1546.81	4662
4600	4466	1547.40	5252
5100	4951	1547.99	5841
5600	5437	1548.58	6432
6100	5922	1549.17	7019

where “deformation” stands for stretching distance of the optical fiber and “strain” is calculated as the ratio of “deformation” to the original length of the optical fiber.

## Data Availability

The datasets generated during and/or analyzed during the current study are available from the corresponding author on reasonable request.
